# Smurf1 and Smurf2 mediated polyubiquitination and degradation of RNF220 suppresses Shh-group medulloblastoma

**DOI:** 10.1038/s41419-023-06025-2

**Published:** 2023-08-03

**Authors:** Yuwei Li, Huishan Wang, Bin Sun, Guifeng Su, Yu Cang, Ling Zhao, Shuhua Zhao, Yan Li, Bingyu Mao, Pengcheng Ma

**Affiliations:** 1 State Key Laboratory of Genetic Resources and Evolution, Kunming Institute of Zoology, Chinese Academy of Sciences, Kunming, 650201 China; 2 Kunming College of Life Science, University of Chinese Academy of Sciences, Kunming, 650203 China; 3 Laboratory of Animal Tumour Models, Frontiers Science Center for Disease-related Molecular Network, West China Hospital, Sichuan University, Chengdu, 610041 China; 4 Key Laboratory of Medicinal Chemistry for Natural Resource, School of Pharmacy, Ministry of Education, School of Pharmacy, Yunnan University, Kunming, 650091 China; 5 Department of Urology, the Affiliated Hospital of Yunnan University, Kunming, 650021 China; 6 Animal Center of Kunming Institute of Zoology, Chinese Academy of Sciences, Kunming, 650201 China; 7 The First Affiliated Hospital of Kunming Medical University, Kunming, 650032 China; 8 Center for Excellence in Animal Evolution and Genetics, Chinese of Academy of Sciences, Kunming, 650201 China

**Keywords:** CNS cancer, CNS cancer, Morphogen signalling

## Abstract

Sonic hedgehog (Shh)-group medulloblastoma (MB) (Shh-MB) encompasses a clinically and molecularly distinct group of cancers originating from the developing nervous system with aberrant high Shh signaling as a causative driver. We recently reported that RNF220 is required for sustained high Shh signaling during Shh-MB progression; however, how high RNF220 expression is achieved in Shh-MB is still unclear. In this study, we found that the ubiquitin E3 ligases Smurf1 and Smurf2 interact with RNF220, and target it for polyubiquitination and degradation. In MB cells, knockdown or overexpression of Smurf1 or Smurf2 promotes or inhibits cell proliferation, colony formation and xenograft growth, respectively, by controlling RNF220 protein levels, and thus modulating Shh signaling. Furthermore, in clinical human MB samples, the protein levels of Smurf1 or Smurf2 were negatively correlated with those of RNF220 or GAB1, a Shh-MB marker. Overall, this study highlights the importance of the Smurf1- and Smurf2-RNF220 axes during the pathogenesis of Shh-MB and provides new therapeutic targets for Shh-MB treatment.

## Introduction

As an aggressive tumor arising from the developing cerebellum, medulloblastoma (MB) is the most common malignant brain tumor in pediatrics [1]. Based on its molecular, clinical, pathological and prognostic characteristics, MB is divided into four groups: the wingless (Wnt) group, the sonic hedgehog (Shh) group, group 3, and group 4 [2, 3]. The classification of MB provides a rationale for exploring molecular-based therapies and prognostics. Despite the survival rate is significantly improved by multimodal treatment regimens, about 30% of MB patients remain incurable [4].

Originating from the cerebellar granule neuron precursor (CGNP) of the external granule cell layer, Shh-group MB (Shh-MB) is characterized by Shh signaling overactivation [1]. Shh signaling is absolutely required for the proliferation of CGNP during cerebellar development [5, 6]. Upon the binding of the Shh ligand to its receptor Ptch1, intracellular signals, including Gli transcription factors, are activated by smoothened (Smo) inhibition release [7, 8] Overactivation of Shh signaling resulting from mutations, including those leading to Ptch1 expression loss or Smo inhibition, leads to CGNP hyperproliferation and thus MB transformation [9]. Overactivated Shh signaling causes approximately 30% of MB cases [10]. Therefore, the physiological and pathological significance of Shh signaling emphasizes the need to fine-tune its action.

Our previous studies illustrated the role of RNF220 in Shh signaling transduction, neural system development and homeostasis [11–13]. During neural patterning, establishment and maintenance of the Shh/Gli gradient are achieved by RNF220-mediated Glis polyubiquitination [11]. During cerebellar development, RNF220 positively regulates Shh signaling through targeting EED, a component of the PRC2 complex, to drive CGNP proliferation. In addition, RNF220-mediated Shh signaling modulation contributes MB cell proliferation both in vitro and in vivo; and the RNF220 protein is overexpressed in *Ptch1*^*±*^ mouse spontaneous orthotopic MB tissues and human clinical Shh-MB samples [12]. Although our previous study suggested that high RNF220 protein expression in Shh-MB is achieved translationally or post-translationally [12], the exact mechanism remains unknown.

Smurf1 and Smurf2 belong to the HECT domain-containing ubiquitin E3 ligase family and regulate multiple pathways, including Wnt, BMP, JNK, and Shh signaling, *via* multiple targets [14]. For instance, during Shh signal transduction, endocytic turnover and clearance of Ptch1 are controlled by Smurf1- and Smurf2-mediated polyubiquitiantion, by which Smurf1 and Smurf2 are required for the Shh-sustained proliferation of CGNP during cerebellar development [15–17]. In addition, Smo is directly targeted by Smurf1 and Smurf2 for polyubiquitination and thus degradation when Shh signaling is off [18]. The role of Smurf1 and Smurf2 in tumorigenesis remains under debate. Both Smurf1 and Smurf2 have been widely reported to function as either promoters or suppressors by regulating various biological processes, including cell proliferation, death, metastasis, senescence and genome stability, in different tumors [14]. Therefore, the role and regulation of Smurf1 or Smurf2 are emerging critical topics in tumor biology research. The expression levels of Smurf1 and Smurf2 are downregulated in Shh-MB [19, 20]; however, the function and mechanism of Smurf1 or Smurf2 during Shh-MB progression remain unknown.

Here, we report that Smurf1 and Smurf2 interact with RNF220 and target it for polyubiquitination and degradation. In MB cells, knockdown or overexpression of Smurf1 or Smurf2 promotes or decreases cell proliferation, respectively, by modulating Shh signaling through RNF220. The Smurf1- and Smurf2-RNF220 axes were validated by the strong negative correlation between Smurf1 or Smurf2 and RNF220 in human clinical MB samples. Therefore, our study expands our understanding on functions of Smurf1 and Smurf2 in cancer progression, highlights the role of the Smurf1- and Smurf2-RNF220 axes during Shh-MB progression, and provides new potential targets for Shh-MB treatment.

## Materials and methods

### Animals

All mice were maintained and handled according to guidelines (IACUC-PA-2023-03-034) approved by the Animal Care and Use Committee of the Kunming Institute of Zoology, Chinese Academy of Sciences. All mice were kept in individual ventilated cages (IVC) in specific pathogen–free (SPF) environment. *Ptch1*^*±*^ mice were gifts from Steven Yan Cheng (School of Basic Medical Sciences, Nanjing Medical University) were genotyped using genomic DNA prepared from tail tips with the following primers: mutant allele, forwards, 5′-CACGGGTAGCCAACGCTATGTC-3′ and reverse, 5′-GCCCTGAATGAACTGCAGGACG-3′; wild-type allele, forwards, 5′-CTGCGGCAAGTTTTTGGTTG-3′ and reverse, 5′-AGGGCTTCTCGTTGGCTA CAAG-3′. BALB/c nude mice were purchased from Charles River Company.

### Cell culture

Human HEK293, Daoy and UW228 cells were from Conservation Genetics CAS Kunming Cell Bank; and were grown in Dulbecco’s Modified Eagle Medium (DMEM) (Gibico) supplemented with 10% fetal bovine serum (FBS) (Gibico), 100 units/mL penicillin (Gibico) and 100 mg/mL streptomycin (Gibico). Cells were transfected with Lipofectamine 2000 (Invitrogen) according to the manufacturer’s instructions for transient expression of the indicated plasmids or siRNAs.

### Lentivirus preparation, infection and stable cell line construction

The following shRNA sequences targeting human Smurf1 and Smurf2 were synthesized and cloned into the lentiviral knockdown vector pLKO.1: shSmurf1: 5′-GCCCAGAGATACGAAAGAGAT-3′, and shSmurf2: 5′-CGGTACAAGTCACATTTCATT-3′. shRNA sequences targeting human RNF220 were used as described in our previous study [12]. Sequences encoding wild-type and ubiquitin E3 ligase-defective mutants of human Smurf1 and Smurf2 were subcloned into the lentiviral overexpression vector pTomo. The lentiviral knockdown or overexpression vectors were cotransfected into HEK293T cells with the lentiviral packaging plasmid pCMV 8.9 and envelope plasmid pMD2.G at a ratio of 10:5:2. At 48 and 72 h after transfection, the cell medium was harvested and centrifuged at 2500 rpm at 4 °C for 10 min, and the supernatant was filtered with 0.45 µm filters. The supernatant containing the recombinant lentivirus was centrifuged at 25,000 rpm at 4 °C for 2.5 h. The lentivirus pellet was then resuspended in pre-cooled PBS containing 0.1% bovine serum albumin (BSA), aliquoted and stored at -80 °C. The copy number of the lentiviral particles was quantified *via* quantitative polymerase chain reaction (qPCR) using the following U5 primers: forwards, 5′-AGCTTGCCTTGAGTGCTTCA-3′ and reverse, 5′-TGACTAAAAGGGTCTGAGGG-3′. Daoy or UW228 cells (5 × 10^5^) were seeded in 6-cm plates and then infected by the recombinant lentivirus at a multiplicity of infection (MOI) of 10. 72 h after infection, the stably transfected cells were selected with puromycin at a concentration of 2 µg/mL for 1 week.

### Plasmids and siRNAs

Plasmids encoding wild-type and truncated mouse RNF220 were used as previously described [11]. Wild-type and KR mutated ubiquitin expression plasmids were gifts from Ceshi Chen’s lab (Kunming Institute of Zoology, Chinese Academy of Sciences). Wild-type and catalytically inactive mutants of human Smurf1 and Smurf2 expression plasmids were gifts from Naihe Jing’s lab (Center for Excellence in Molecular Cell Science, Chinese Academy of Sciences). Site-directed mutations were conducted by PCR-driven overlapping extension using high-fidelity *Pfu* DNA polymerase (Fermentas) to prepare the RNF220 KR and SA mutated constructs. Control siRNA and siRNAs targeting human Smurf1 and Smurf2 were synthesized by RIBOBIO Company as follows: si-Control: 5′-AATTCTCCGAACGTGTCACGT-3′, si-Smurf1: 5′-AACCTTGCAAAGAAAGACTTC-3′, and si-Smurf2: 5′-GCAGTTAATCCGGAACATTTA-3′.

### RNA isolation, cDNA synthesis and quantitative real-time PCR

Total RNA was isolated from tissues and cultured cells with TRIzol reagent (TianGen) according to the manufacturer’s instructions. 1 μg of total RNA was used for first-strand cDNA synthesis through reverse transcription using a cDAN synthesis kit (Fermantas). Expression level of target genes was quantified using the LightCycler 480 SYBR Green I Master (Roche) on a LightCycler 480 system (Roche). All reactions were run at least in triplicate. Primers used were described as follows: mouse *Smurf1* forwards, 5′-CTACCAGCGTTTGGATCTAT-3′ and reverse, 5′-TTCATGATGTGGTGAAGCCG-3′; mouse *Smurf2* forwards, 5′-TAAGTCTTCAGTCCAGAGACC-3′ and reverse, 5′-AATCTCTTCCCTAGACACCTC-3′; human *Smurf1* forwards, 5′-CTGTACTGGACCACACCTTCTG-3′ and reverse, 5′-CTAAGAACTGGGCTTCGATTC-3′; and human *Smurf2* forwards, 5′-CATACACAGACTGGTGTGAGC-3′ and reverse, 5′-GTATTACGGATCTCCCATCCAG-3′. Primers for mouse and human *Actin* and *RNF220*, human *Gli1*, *Ptch1* and *Hhip1* were used as previously described [12].

### Co-immunoprecipitation (co-IP) and western blot (WB) assays

Cells were lysed in immunoprecipitation (IP) lysis buffer [50 mM Tris-HCl, pH 7.5, 150 mM NaCl, 1% Triton X-100 and protease inhibitors cocktail (ThermoFisher Scientific)], and then lysates were clarified by centrifugation for 15 min at 14,000 rpm at 4^o^C. The protein concentration of each lysate was determined using a bicinchoninic acid assay (BCA) protein assay kit according to the manufacturer’s instructions (ThermoFisher Scientific). IP was carried out with anti-Flag, anti-Myc, anti-Smurf1 or anti-Smurf2 antibodies coupled with protein A and G agarose beads (SantaCruz). Then, the original input and isolated proteins were subjected to WB assays followed by immunoblotting with the following antibodies: anti-Tubulin (66031-1, Proteintech, 1:5000), anti-Myc (C3956, Sigma‒Aldrich, 1:5000), anti-HA (H3663, Sigma‒Aldrich, 1:5000), anti-Flag (F7425, Sigma‒Aldrich, 1:5000), anti-RNF220 (HPA027578, Sigma‒Aldrich, 1:2000), anti-ubiquitin (sc-8017, SantaCruz, 1:1000), anti-Smurf1 (2174, Cell Signaling Technology, 1:2000), anti-Smurf2 (12024, Cell Signaling Technology, 1:2000), anti-Gli1 (2534, Cell Signaling Technology, 1:2000), anti-Ptch1 (17520-1-AP, Proteintech, 1:2000), and anti-Hhip1 (11654-1-AP, ThermoFisher Scientific, 1:2000). Horseradish peroxidase (HRP)-coupled goat anti-mouse or rabbit IgG (ThermoFisher Scientific) was used as the secondary antibody. Chemiluminescence detection was conducted using a chemiluminescent protein detection kit (ThermoFisher Scientific) according to the manufacturer’s instructions.

### Ubiquitination assays

For the in vivo ubiquitination assays, HEK293 cells were transfected with the indicated plasmids and were harvested at 48 h later. Transfected cells were treated with 25 nM MG132 (MedChem Express), a proteasomal inhibitor, for 6 h prior to harvesting. Harvested cells were lysed in a SDS lysis buffer [50 mM Tris-HCl pH 6.8, 1.5% SDS, and protease inhibitors cocktail (ThermoFisher Scientific)] for 15 min at 95 °C. Following 10-fold dilution of the lysate with a extraction buffer [50 mM Tris-HCl pH 6.8, 180 mM NaCl, 0.5% NP-40, 0.5% BSA, and protease inhibitors cocktail (ThermoFisher Scientific)], the cell lysates were then immunoprecipitated by anti-Flag M2 beads (A2220, Sigma‒Aldrich). The bound proteins were eluted with 1× Laemmli sample buffer (A3401, Sigma-Aldrich) at 95 °C for 10 min. WB assays were conducted using an anti-HA or anti-ubiquitin antibody.

The in vitro ubiquitination assays were performed in a 50 μL reaction volume containing the following components: 5 mg wild-type ubiquitin (Boston Biochem), 0.2 mg E1 (Boston Biochem), 0.2 mg E2 (UbcH5h, Boston Biochem), 1 mg purified wild-type or KR mutated RNF220 protein, 1 mg purified wild-type or E3 ubiquitin ligase inactive Smurf1 or Smurf2 protein (purified with anti-Flag or anti-Myc beads from HEK293 cells), 5 μL 10 × reaction buffer (Boston Biochem), 2 mM ATP (Cell Signalling Technology) and 5 mM MgCl_2_ (Sigma-Aldrich). Reactions were incubated at 30 °C for 1 h terminated by boiling at 95 °C for 10 min with 1 × Laemmli sample buffer (A3401, Sigma-Aldrich) and then processed for WB assays with the antibodies indicated.

### Mice xenograft

A total of 2 × 10^6^ indicated Daoy cells resuspended in PBS with 20% Matrigel (356237, Corning) were subcutaneously injected into 8-week-old female BALB/c nude mice (*n* = 6 for each group). There were six mice per group. Mice were killed 8 weeks later and the xenograft size was measured by the formula: 0.5 × length × width^2^.

### MTS assay

The indicated Daoy or UW228 cells (1 × 10^3^) were seeded in 96-well plates. At the following indicated time points, 20 µL MTS reagent (G3582, Promega) was added to each sample, and reactions were incubated at 37 °C for 1 h. Then, the optical density (OD) value at 492 nm was measured using a microplate reader (Bio-Rad Laboratories). The data are showed as the mean ± standard deviation (SD).

### Soft agar assay

Complete cell culture medium containing 0.6% low-melting point agar (39346-81-1, Sigma‒Aldrich) was mixed by pipetting gently and quickly aliquoted at 1 mL/well in a 6-well plate. Then, the plates were cooled for approximately 15 min at 4 °C to solidify the agar, which was used as the base agar. Total of 2 × 10^4^ Daoy or UW228 cells were mixed with 1 mL complete cell culture medium containing 0.3% low-melting point agar and quickly layered on the top of the pre-cooled base agar. To allow the upper agar solidification, the plates were then placed in a 37^o^C with 5% CO_2_ incubator for 30 min. Finally, 1 mL complete cell culture medium was layered on the top of the upper agar and cells were cultured for 4 weeks. 1 mL of PBS containing 4% formaldehyde (PFA) and 0.005% crystal violet was added to fix and stain the colonies, and images were counted, captured and analyzed using an inverted phase contrast microscope (IX73, Olympus).

### EdU assay

To analyze cell proliferation, Daoy or UW228 cells were incubated with 10 μM 5-ethyl-20-deoxyuridine (EdU) for 1 h, and then were fixed with 4% PFA followed by permeabilization with 0.3% Triton X-100 in PBS. An EdU assay kit (Beyotime Biotechnology) was used for the subsequent staining. Staining images were captured, and analyzed using an epifluorescence microscope (IX73, Olympus).

### Immunohistochemical (IHC) staining

Paraffin-embedded clinical human MB specimens were obtained from Bioaitech Company (N035Cb01). Immunohistochemical staining assays were conducted as described [12]. The antibodies used were listed as follows: anti-GAB1 (GTX111253, 1:50, GeneTex), anti-RNF220 (HPA027578, 1:200, Sigma‒Aldrich), anti-Smurf1 (2174, 1:200, Cell Signaling Technology), and anti-Smurf2 (12024, 1:200, Cell Signaling Technology). Images were captured and analyzed using an epifluorescence microscope (IX73, Olympus).

### Statistical analysis

Each experiment was conducted at least three times independently. Statistical analysis for real-time RT-PCR, EdU staining, cell proliferation, cell colony formation, and tumor xenografts assays, quantitive data were collected from the indicated experiment shown in figures. Statistical analysis for WB and immunohistochemical staining assays, quantitive data were collected from all independently repeated experiments. Not any data were excluded from all the analyzes. Statistical significances were performed using the GraphPad Prism software (GraphPad Software Inc., La Jolla, CA, USA). For experiments including real-time RT‒PCR, EdU staining, and colony formation, statistical difference was evaluated using the two-tailed Student’s *t-test* for the comparison between two experimental groups. For association analyzes between gene expression levels, statistical significance was assessed by Pearson product-moment correlation coefficient analysis. A *P* values < 0.05 was considered as statistically significance (*), and <0.01 was regarded as statistically very significant (**). The WB and IHC staining data were quantified by ImageJ software (National Institute of Health).

## Results

### Smurf1 and Smurf2 interact with RNF220

**Fig. 1:**
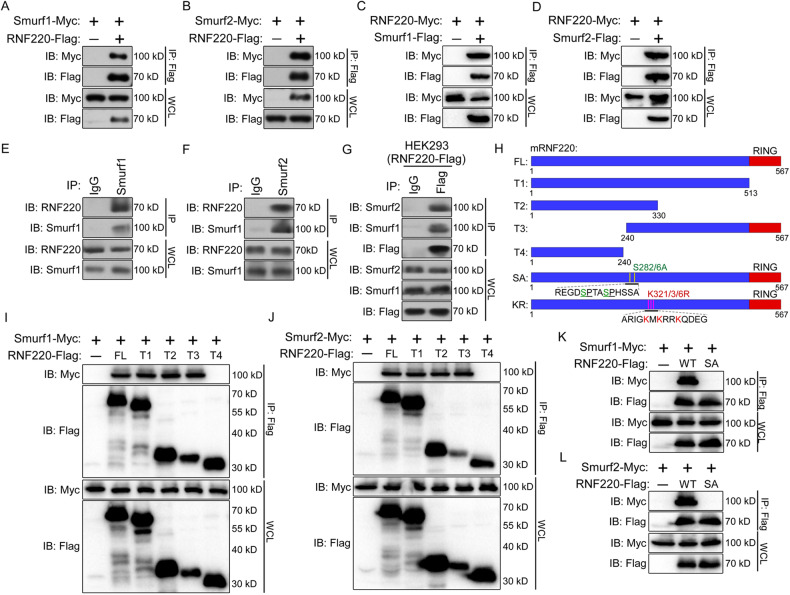
RNF220 interacts with Smurf1 and Smurf2. **A, B** Smurf1 (**A**) or Smurf2 (**B**) was co-immunoprecipitated with RNF220. **C, D** RNF220 was co-immunoprecipitated with Smurf1 (**C**) or Smurf2 (**D**). HEK293 cells were transiently transfected with different combinations of expression vectors of Smurf1, Smurf2, and RNF220, as indicated. Cell lysates were incubated with anti-Flag beads, washed, and subsequently analyzed by Western Blot. **E, F** Endogenous RNF220 could be immunoprecipitated by Smurf1 (**E**) or Smurf2 (**F**). Cell extracts of HEK293 cells were immunoprecipitated with antibody against Smurf1 or Smurf2, and endogenous RNF220 was detected by an anti-RNF220 antibody, with IgG as a negative control. **G** Endogenous Smurf1 and Smurf2 could be immunoprecipitated by RNF220. Cell extracts of HEK293 cells stably transfected with RNF220-Flag vectors were immunoprecipitated with anti-Flag beads, and endogenous Smurf1 or Smurf2 was detected by an anti-Smurf1 or anti-Smurf2 antibody, with IgG as a negative control. **H**–**L** The ability of different RNF220 deletion constructs or mutants to pull down Smurf1 (**I**, **K**) or Smurf2 (**J**, **L**) in co-immuniprecipitation assays. Schematic representation of the structures of truncated or mutated mouse RNF220 with amino acid numbers indicated is shown in (**H**). IB immunoblot, IP immunoprecipitation, WCL whole cell lysate, WT wild-type, FL full-length, SA RN220^S282/286A^ mutant, and KR RNF220^K321/323/326R^ mutant.

Our previous observation suggested that the RNF220 protein is stabilized in Shh-MB through a posttranslational mechanism [12]. The results of our previous yeast two-hybrid (Y2H) screening indicated that Smurf1 and Smurf2, two ubiquitin E3 ligases, might be potential candidates that directly control the stability of the RNF220 protein [21, 22]. Firstly, co-immunoprecipitation (co-IP) experiments were carried out to confirm the binding between RNF220 and Smurf1 or Smurf2. When cotransfected in HEK293 cells, either Smurf1 or Smurf2 associated with RNF220 (Fig. 1A–D). To further confirm that their binding was not spurious due to overexpression, we isolated endogenous RNF220, Smurf1, or Smurf2 from HEK293 cells. Endogenous RNF220 was detected in both the Smurf1 and Smurf2 immunoprecipitates (Fig. 1E, F). When we isolated RNF220 using an anti-Flag beads from HEK293 cells stably transfected with the RNF220-Flag construct, both endogenous Smurf1 and Smurf2 were also pulled down together (Fig. 1G).

To identify the corresponding domains involved in the aforementioned interaction, a series of RNF220 truncated constructs were tested in co-IP experiments (Fig. 1H–J). Deletion of the C-terminal RING domain or N-terminal 240 amino acids of RNF220 did not affect RNF220 association with Smurf1 or Smurf2. In addition, the fragment containing the RNF220 N-terminal 330 amino acids, but not that containing the N-terminal 240 amino acids, could still interact with Smurf1 or Smurf2 (Fig. 1H–J). These results suggest that the fragment containing amino acids 240-330 is crucial for the interaction between RNF220 and Smurf1 or Smurf2. Smurf1 and Smurf2 belong to the HECT-type ubiquitin E3 ligase family and are endowed with WW domains that mediate interactions with substrates. These interactions are mediated by specific WW-docking sites, such as PPXY and phospho-serine/proline motifs [23–27]. Interestingly, there are two phospho-serine/proline motifs in the fragment containing amino acids 240-330 of RNF220 (Fig. 1H). To test whether these motifs are involved in the binding between RNF220 and Smurf1 or Smurf2, we disrupted both consensus sequences by replacing serine with alanine at residues 282 and 286 of full-length RNF220 (RNF220^S282/6A^). As shown in Fig. 1K, L, RNF220^S282/6A^ failed to bind Smurf1 or Smurf2, implying that pS^282^P and pS^286^P represent the specific Smurf1- or Smurf2-binding sites of RNF220, respectively.

### Smurf1 and Smurf2 regulate RNF220 protein stability

**Fig. 2:**
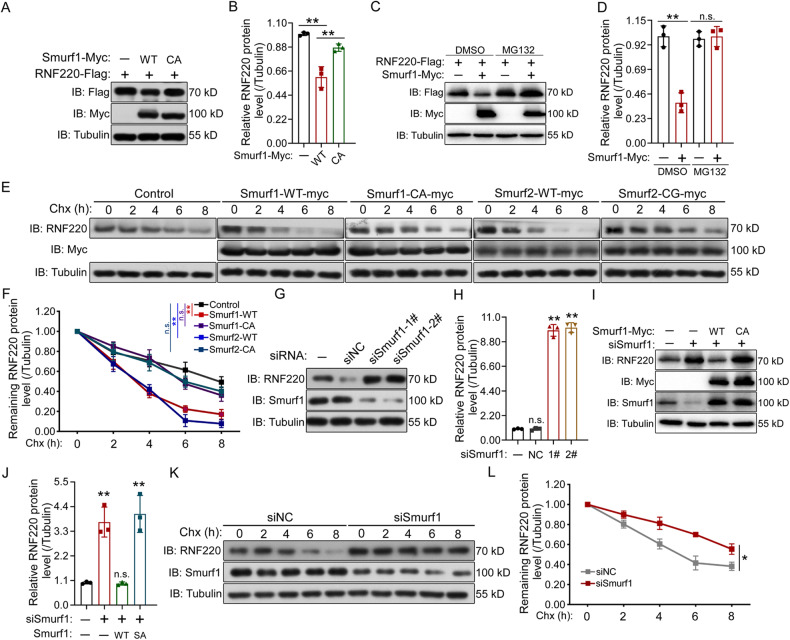
Smurf1 regulates RNF220 protein stability. **A, B** RNF220 protein was destabilized by overexpression of wild-type Smurf1 (**A**), but not their E3 ubiquitin ligase-defective mutants. Flag-tagged RNF220, myc-tagged wild-type or ligase-defective Smurf1 plasmids were transfected into HEK293 cells as indicated. After 48 h, cell lysates were analyzed by Western Blot. **B** Bar graphs, overlaid with the actual data points, show relative RNF220 protein expression (mean ± SD) normalized against the corresponding α-Tubulin. The control was set to 1. **C, D** Western Blot assays showing the protein level of RNF220 when co-expressed with Smurf1 in presence of MG132 or not. **D** Bar graphs, overlaid with the actual data points, show relative RNF220 protein expression (mean ± SD) normalized against the corresponding α-Tubulin. The respective controls were set to 1. **E, F** Effects of wild-type or ligase-defective Smurf1 or Smurf2 overexpression on the protein stability of RNF220. HEK293 cells were transiently transfected with the indicated plasmids. At 48 h posttransfection, cycloheximide was added to all samples, and cells were then harvested at the time points indicated. Level of endogenous RNF220 was determined by Western Blot with an anti-RNF220 antibody. In all cases, α-Tubulin was used as a loading control. The relative levels of RNF220 were quantified densitometrically and normalized against α-Tubulin. The data in **F** are the average of three independent experiments. **G, H** Western Blot results showing the effects of Smurf1 or Smurf2 knockdown on RNF220 protein level in HEK293 cells. HEK293 cells were transfected with the indicated siRNAs, and 72 h later, cells were harvested for Western Blot analysis. **H** Bar graphs, overlaid with the actual data points, show the relative expression (mean ± SD) of RNF220, normalized against the corresponding α-Tubulin. The control was set to 1. **I, J** Western Blot analysis showing the protein level of endogenous RNF220 when wild-type or E3 ubiquitin ligase activity defective Smurf1 was co-expressed with siRNAs against Smurf1 in HEK293 cells. The statistics of the result was shown in (**J**) with α-Tubulin as a loading control, and the control was set to 1. **K, L** Effect of Smurf1 knockdown on the protein stability of endogenous RNF220 in HEK293 cells. Cells were transiently transfected with the indicated siRNAs. At 72 h posttransfection, cycloheximide was added to all samples, and the cells were then harvested at the time points indicated. Protein level of RNF220 was determined by Western Blot with an anti-RNF220 antibody. The relative levels of RNF220 were quantified densitometrically and normalized against α-Tubulin. **L** The statistics showing the average of three independent experiments. IB immunoblot, WT wild-type, CA Smurf1 E3 ubiquitin ligase-defective mutant, CG Smurf2 E3 ubiquitin ligase-defective mutant, NC negative control, Chx cycloheximide, ns not significant. *p* > 0.05, *p < 0.05, **p < 0.01.

Given that RNF220, Smurf1, and Smurf2 are all ubiquitin E3 ligases that often regulate the stability of their targeted proteins, we first examined whether the level of either Smurf1 or Smurf2 protein was regulated by RNF220 overexpression. The results showed that the protein levels of Smurf1 and Smurf2 were not affected when co-expressed with wild-type RNF220 or its catalytic RING domain–deleted truncated isoform in HEK293 cells (Supplementary Fig. 1A, B). We next determined whether Smurf1 or Smurf2 regulates RNF220 protein levels and found that overexpression of wild-type Smurf1 or Smurf2, but not their ubiquitin E3 ligase-defective mutants (Smurf1-CA or Smurf2-CG) [28, 29], reduced RNF220 protein levels (Fig. 2A, B and Supplementary Fig. 1C, D). MG132, a proteasome inhibitor, blocked the Smurf1 or Smurf2 overexpression-induced decrease in RNF220 protein levels (Fig. 2C, D and Supplementary Fig. 1E, F). We then used cycloheximide (Chx)-based protein chasing assays to test the stability of RNF220 protein co-expressed with wild-type or ubiquitin E3 ligase-defective mutants of Smurf1 or Smurf2. Quantification of the steady-state protein levels showed faster degradation of RNF220 in the presence of wild-type Smurf1 or Smurf2, but not their E3 ubiquitin ligase-defective mutants (Fig. 2E, F). To further test the function of Smurf1 and Smurf2 regarding RNF220, we performed knockdown experiments using two independent Smurf1 or Smurf2 siRNAs in HEK293 cells. The siRNAs for Smurf1 and Smurf2 reduced their endogenous expression by over 70% the mRNA (Supplementary Fig. 1G, H) and protein levels (Fig. 2G, H and Supplementary Fig. 1I, J). Immunoblotting results showed that the protein level of RNF220 was increased when either Smurf1 or Smurf2 was knocked down (Fig. 2G, H and Supplementary Fig. 1I, J). To rule out the off-target effects of Smurf1 or Smurf2 siRNAs, we re-expressed wild-type or their ubiquitin E3 defective mutants in knockdown cells. Wild-type Smurf1 or Smurf2, but not their ubiquitin E3 ligase-defective mutants, obviously reversed the upregulation of RNF220 protein levels mediated by Smurf1 or Smurf2 knockdown (Fig. 2I, J and Supplementary Fig. 1K, L). In addition, as expected, Smurf1 or Smurf2 knockdown significantly increased the protein stability of the RNF220 protein, as indicated by protein chase assays (Fig. 2K, L and Supplementary Fig. 1M, N). Notably, the level of RNF220 mRNA transcript was not affected by Smurf1 or Smurf2 knockdown in HEK293 cells (Supplementary Fig. 1O). Collectively, these results indicate that Smurf1 and Smurf2 regulate the stability of RNF220 protein in a manner that is dependent on their ubiquitin E3 ligase activity.

### Smurf1 and Smurf2 target RNF220 for polyubiquitination

**Fig. 3:**
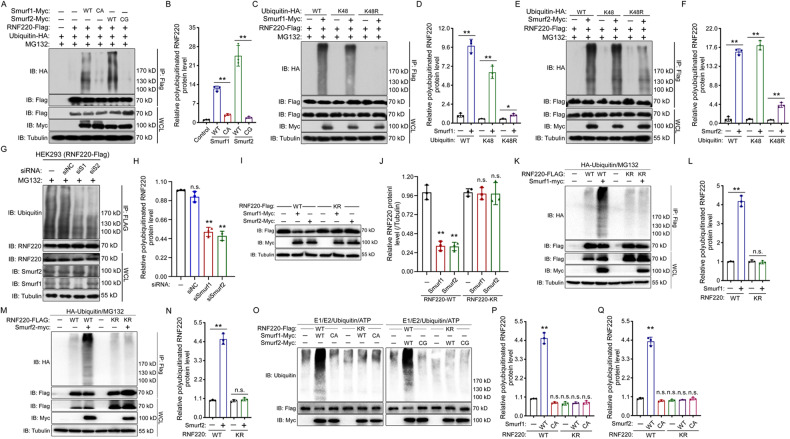
Smurf1 and Smurf2 target RNF220 for K48-linked polyubiquitination. **A, B** In vivo ubiquitination assays showing the ubiquitination status of RNF220 when wild-type or E3 ubiquitin ligase inactive form of Smurf1 or Smurf2 was co-expressed in HEK293 cells. Overexpression of wild-type Smurf1 or Smurf2, but not their mutants promoted the ubiquitination of RNF220. The statistics of relative protein levels of polyubiquitinated RNF220 in each group was shown in (**B**), and the control group was set to 1. **C**–**F** In vivo ubiquitination assays showing the ability of Smurf1 (**C**, **D**) or Smurf2 (**E**, **F**) to ubiquitinate RNF220 when the indicated ubiquitin mutants were used. Smurf1 and Smurf2 promote K48-linked ubiquitination of RNF220. For ubiquitin mutants: K48 the K48 ubiquitin mutant with all lysines except the K48 mutated to arginines, K48R the K48R ubiquitin mutant with only the K48 lysine mutated to arginine, WT the wild-type ubiquitin construct. The statistics of relative protein levels of polyubiquitinated RNF220 in each group was shown in (**D**, **F**), and the control group was set to 1. **G, H** In vivo ubiquitination analysis showing the level of endogenous ubiquitinated RNF220 protein when Smurf1 or Smurf2 was knocked down in HEK293 cells by siRNA transfection. The statistics of relative protein levels of ubiquitinated RNF220 in each group was shown in (**H**), and the control group was set to 1. **I, J** Western blot analysis showing the protein level of wild-type or KR mutated form of RNF220 protein when co-expressed with Smurf1 or Smurf2 in HEK293 cells. The statistics of relative protein levels of RNF220 in each group was shown in (**J**), and the respective controls were set to 1. **K**–**N** In vivo ubiquitination analysis showing the ubiquitinated status of wild-type or KR mutated form of RNF220 protein when co-expressed with Smurf1 (**K**, **L**) or Smurf2 (**M**, **N**). **O**–**Q** In vitro ubiquitination analysis showing that Smurf1 or Smurf2 promotes polyubiquitination of wild-type, but not KR mutated, RNF220 protein. The statistics of relative protein levels of RNF220 in each group was shown in (**P**, **Q**), and the respective controls were set to 1. IB immunoblot, IP immunoprecipitation, WCL whole cell lysate, WT wild type, KR RNF220^K321/323/326R^ mutant; CA Smurf1 E3 ubiquitin ligase-defective mutant, CG Smurf2 E3 ubiquitin ligase-defective mutant; NC negative control, ns not significant. *p* > 0.05 and **p < 0.01.

The above data suggest that RNF220 might be a direct target for Smurf1 and Smurf2; therefore, we carried out ubiquitination assays to test this hypothesis. We demonstrated that coexpression of wild-type Smurf1 or Smurf2 significantly upregulated the level of polyubiquitinated RNF220 protein, whereas the catalytically inactive Smurf1-CA or Smurf2-CG mutants did not (Fig. 3A, B). Ubiquitin ligases can target their substrates for different types of polyubiquitin chains, including K6, K11, K27, K29, K33, K48, and K63, with different functional effects [30]. Usually, K48-linked polyubiquitination target proteins for proteasomal degradation, while the others have mostly been implicated in nonproteolytic regulation [31, 32]. Thus, to characterize the type of ubiquitin chains added by Smurf1 or Smurf2 to RNF220, different ubiquitin mutants, either all lysines of ubiquitin except K48 were substituted by arginine (K48) or only K48 was substituted by arginine (K48R), were tested in ubiquitination assays. The results showed that Smurf1 and Smurf2 target RNF220 for K48-linked polyubiquitination, which is consistent with the destabilization effect of Smurf1 and Smurf2 on the RNF220 protein (Fig. 3C–F).

To further examine whether RNF220 is targeted by endogenous Smurf1 or Smurf2 for polyubiquitination, we conducted ubiquitination assays in Smurf1 or Smurf2 knockdown cells. The results showed that polyubiquitinated RNF220 levels were reduced in both Smurf1- and Smurf2-knockdown cells (Fig. 3G, H), suggesting that endogenous Smurf1 and Smurf2 play a role in RNF220 polyubiquitination in HEK293 cells. Our previous study demonstrated that lysines 321, 323, and 326 serve as potential polyubiquitination sites responsible for RNF220 protein stability controlling [33]. Here, we tested whether the three lysines are responsible for Smurf1- or Smurf2-mediated RNF220 protein stability regulation through polyubiquitination. First, we found that when the three lysines were mutated to arginines (RNF220^KR^), the RNF220 protein level was not sensitive to either Smurf1 or Smurf2 overexpression (Fig. 3I, J). Indeed, when co-expressed with Smurf1 or Smurf2 in HEK293 cells, the polyubiquitination of RNF220^KR^ decreased greatly compared with that of the wild-type RNF220 (Fig. 3K–N). Further, in vitro ubiquitination assays were conducted to confirm these observations using purified RNF220, Smurf1 and Smurf2 proteins. The results showed that wild-type Smurf1 or Smurf2, but not their ligase inactive mutants, efficiently promoted polyubiquitination of RNF220 in vitro (Fig. 3O–Q).

### Smurf1 and Smurf2 modulate Shh signaling through RNF220 in Shh-MB cells

**Fig. 4:**
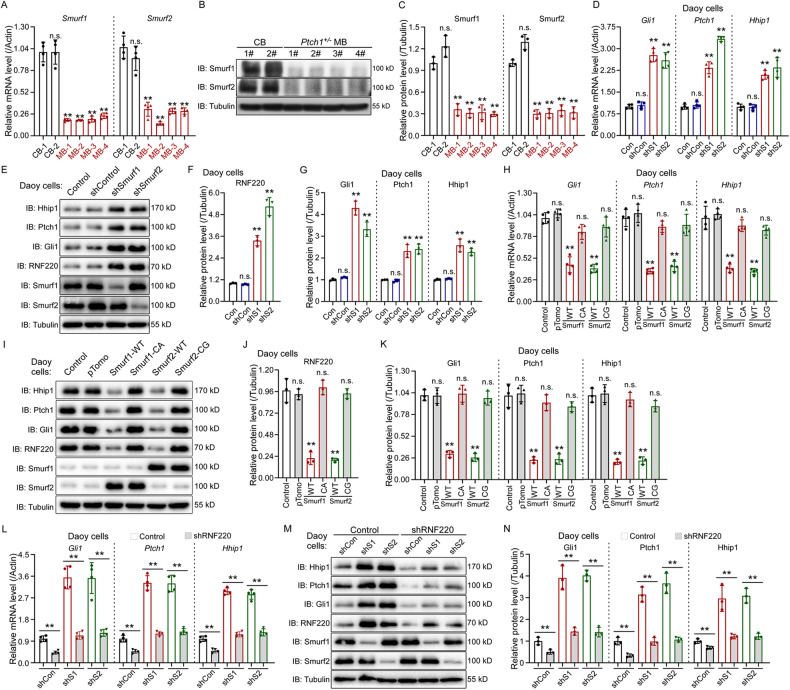
Smurf1 and Smurf2 regulate Shh signaling through RNF220 in Daoy cells. **A**–**C** Real-time RT-PCR (**A**) Western Blot (**B**, **C**) assays showing Smurf1 and Smurf2 expression in control cerebellum and *Ptch1*^*±*^ medulloblastoma tissues. The statistics showing relative Smurf1 and Smurf2 protein levels normalized against the corresponding α-Tubulin level was shown in (**C**), and the respective controls were set to 1. **D**–**G** Real-time RT-PCR (**D**) and Western Blot (**E**–**G**) assays showing the expression levels of RNF220 and Shh targets, including Gli1, Ptch1, and Hhip1, in Daoy cell stably transfected with shRNAs against Smurf1 or Smurf2. The statistics showing the relative protein levels of RNF220, Gli, Ptch1, and Hhip1, normalized against the corresponding α-Tubulin level was shown in (**F**, **G**), and the respective controls were set to 1. **H**–**K** Real-time RT-PCR (**H**) and Western Blot (**I**–**K**) assays showing the expression levels of RNF220 and Shh targets, including Gli1, Ptch1, and Hhip1, in Daoy cells transfected with expression plasmids for wild-type or E3 ubiquitin ligase-defective form of Smurf1 or Smurf2. The statistics showing the relative protein level of RNF220 (**J**), Gli, Ptch1, and Hhip1 (**I**) normalized against the corresponding α-Tubulin level. The respective controls were set to 1. **L**–**N** Real-time PCR (**L**) and Western blot (**M**, **N**) assays showing the expression level of Shh targets, including Gli1, Ptch1, and Hhip1, in Daoy cells stably transfected with the indicated shRNAs against RNF220, Smurf1, or Smurf2. The statistics showing the relative protein levels of Gli, Ptch1, and Hhip1 normalized against the corresponding α-Tubulin level was shown in (**N**). The respective controls were set to 1. β-Actin was used as a loading control for real-time RT-PCR assays. IB immunoblot, CB cerebellum, MB medulloblastoma, ns not significant. *p* > 0.05, **p < 0.01.

Both Smurf1 and Smurf2 were reported to be downregulated in human Shh-MB samples [19, 20]. Here, we confirmed the reduction in Smurf1 and Smurf2 in the *Ptch1*^*±*^ mouse spontaneous orthotopic MB model at both the mRNA and protein levels (Fig. 4A–C).

Our previous study revealed that RNF220 protein was required for sustained Shh activation and upregulated in Shh-MB [12]. We next tested the potential involvement of Smurf1 and Smurf2 in regulating RNF220 protein levels and, thus, Shh signaling in Daoy and UW228 cells, both of which represent Shh-MB [34], *via* shRNA-mediated knockdown and overexpression assays. We firstly confirmed the interaction between RNF220 and Smurf1 or Smurf2 by in vivo co-IP assays in Daoy and UW228 cells (Supplementary Fig. 2). The shRNA knockdown efficiency of Smurf1 or Smurf2 was demonstrated by real-time RT‒PCR in stably transfected Daoy or UW228 cells (Supplementary Fig. 3A, B). Elevated RNF220 protein levels were observed when either Smurf1 or Smurf2 was knocked down in Daoy or UW228 cells (Fig. 4E, F and Supplementary Fig. 3D, E). Conversely, the protein level of RNF220 was decreased in Smurf1- or Smurf2-overexpressing Daoy or UW228 cells (Fig. 4I, J and Supplementary Fig. 3H, I). Additionally, the level of RNF220 mRNA transcript was not affected by Smurf1 or Smurf2 knockdown or overexpression in both cells (Supplementary Fig. 3K, L), implying that regulation occurs at the post-transcriptional level.

To prove the potential role of Smurf1 and Smurf2 in Shh signaling in Daoy and UW228 cells, levels of the direct targets of Shh signaling, including Gli, Ptch1, and Hhip1, were examined in Daoy and UW228 cells with Smurf1 or Smurf2 knockdown or overexpression (Fig. 4D–K and Supplementary Fig. 3C–J). The results showed that all the examined Shh signaling targets, Gli, Ptch1, and Hhip1, were increased in Smurf1- or Smurf2-knockdown cells (Fig. 4D, E, G and Supplementary Fig. 3C, D, F) and decreased in Smurf1- or Smurf2-overexpressing cells (Fig. 4H, I, K and Supplementary Fig. 3G, H, J), suggesting that Shh signaling was modulated by Smurf1 and Smurf2 in MB cells. To demonstrate that Smurf1- and Smurf2-mediated Shh signaling modulation is dependent on their capability to degrade RNF220, we tested the effects of RNF220 knockdown in Smurf1 or Smurf2 knockdown cells on Shh signaling. The results showed that RNF220 knockdown alleviated the upregulation of Shh target genes by Smurf1 or Smurf2 knockdown in both cell lines (Fig. 4L–N and Supplementary Fig. 3M–O). Together, these data suggest that endogenous Smurf1 and Smurf2 inhibit Shh signaling partially through RNF220.

### Smurf1 and Smurf2 knockdown promote Shh-MB cell proliferation

**Fig. 5:**
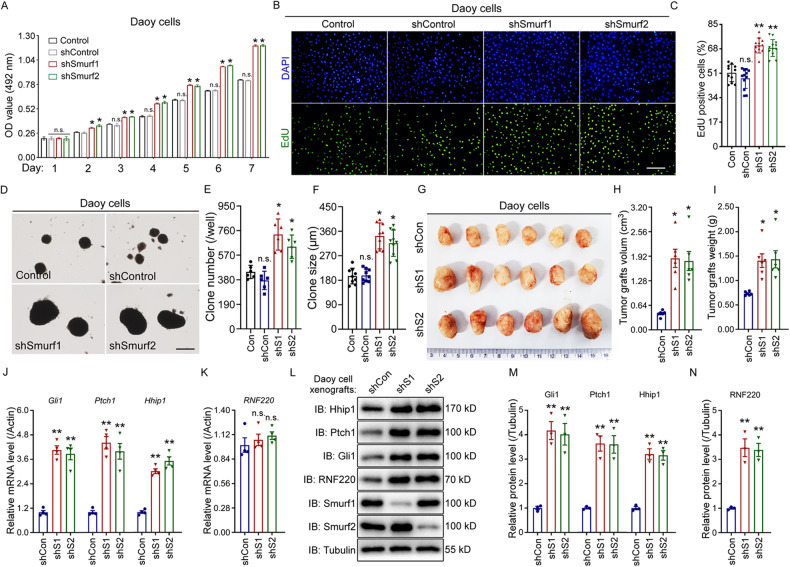
Smurf1 or Smurf2 knockdown accelerates cell proliferation and tumor growth in Daoy cells. **A** Growth curve for control, Smurf1 or Smurf2 knockdown Daoy cell line revealed by MTS assays. **B, C** EdU incorporation assays to evaluate DNA synthesis and proliferation rate of Daoy cells when Smurf1 or Smurf2 was knocked down. Scale bar, 50 µm. Quantification of EdU assay result was shown in (**C**). **D**–**F** Soft agar colony formation assays for the indicated Daoy cell line. Scale bar, 120 µm. Quantification of colony number and size was shown in (**E**, **F**). **G**–**I** Xenograft tumors from BALB/c nude mice subcutaneously injected with Daoy cells stably transfected with shRNAs against Smurf1 or Smurf2. Quantification of xenograft was shown in (**H**) for tumor size and (**I**) for tumor weight. **J**–**N** The mRNA (**J**, **K**) and protein expression (**L**–**N**) level of Shh targets (**J**, **L**, and **M**) and RNF220 (**K**, **L**, and **N**) in the indicated tumor xenografts. S1 Smurf1, S2 Smurf2, IB immunoblot, ns not significant *p* > 0.05, *p < 0.05 and **p < 0.01.

Next, we examined the role of Smurf1 or Smurf2 in Shh-MB cell proliferation, and growth curves were generated for Smurf1 or Smurf2 knockdown Daoy and UW228 cells and compared to controls in vitro. Growth curves generated over 7 days revealed that knockdown of Smurf1 or Smurf2 significantly accelerated the proliferation of Shh-MB cells relative to controls (Fig. 5A and Supplementary Fig. 4A). Increased EdU incorporation in Shh-MB-knockdown cells further demonstrated that Smurf1 or Smurf2 knockdown promoted Shh-MB cell proliferation in vitro (Fig. 5B, C and Supplementary Fig. 4B, C). We then examined the colony formation capacity of cells stably transfected with Smuf1 or Smurf2 shRNA, and the results showed that Smurf1 or Smurf2 knockdown enhanced colony formation capacity in the two cell lines (Fig. 5D–F and Supplementary Fig. 4D–F).

We further examined tumor growth in vivo. Smurf1- or Smurf2-depleted Daoy cells were subcutaneously injected into immune-deficient mice. During a 2-month period, Smurf1 and Smurf2 knockdown cells grew significantly faster than the control cells (Fig. 5G). The average tumor weights and volumes were significantly larger than those of the control xenografts at Day 60 (Fig. 5G–I). We also tested the expression levels of RNF220 and Shh targets in xenografts (Fig. 5J–N). The mRNA and protein expression levels of Shh targets, including Gli1, Ptch1 and Hhip1, were increased in both Smurf1 and Smurf2 knockdown xenografts (Fig. 5J, L, and M). Notably, the protein, but not mRNA, level of RNF220 was upregulated in Smurf1 and Smurf2 knockdown xenografts (Fig. 5K, L, and N). Taken together, these results demonstrate that Smurf1 and Smurf2 knockdown promotes Shh-MB tumorigenesis.

### The ubiquitin E3 ligase activity is required for Smurf1- and Smurf2-mediated inhibition of Shh-MB cell proliferation

**Fig. 6:**
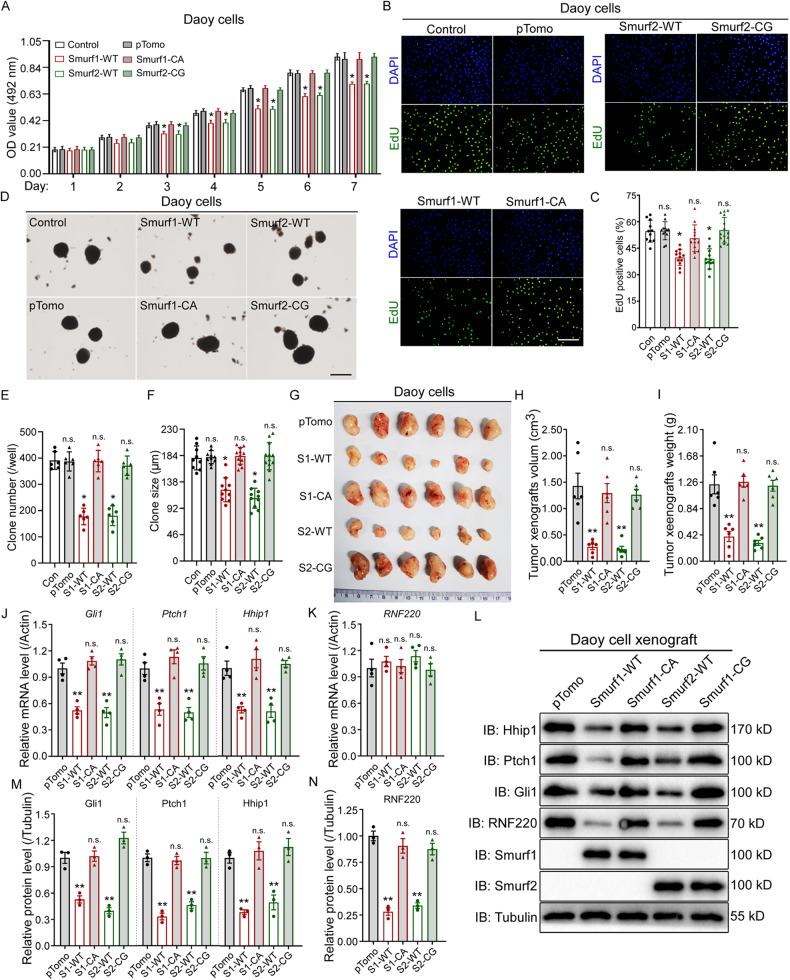
E3 ubiquitin ligase activity is required for cell proliferation and tumor growth inhibition by Smurf1 or Smurf2 overexpression in Daoy cells. **A** Growth curve for control, wild-type or E3 ubiquitin ligase-defective Smurf1 or Smurf2 overexpressed Daoy cell line, revealed by MTS assays. **B, C** EdU incorporation assay to evaluate DNA synthesis and proliferation rates of UW228 cells when wild-type or E3 ubiquitin ligase-defective Smurf1 or Smurf2 was overexpressed. Scale bar, 50 µm. Quantification of the EdU assay results was shown in (**C**). **D**–**F** Soft agar colony formation assays for the indicated Daoy cell line. Scale bar, 120 µm. Quantification of the colony number and size was shown in (**E**, **F**). **G**–**I** Xenograft tumors from BALB/c nude mice subcutaneously injected with wild-type or E3 ubiquitin ligase inactive Smurf1 or Smurf2 overexpressed Daoy cells. Quantification of the xenografts was shown in (**H**) for tumor size and (**I**) for tumor weight. **J**–**N** The mRNA (**J**, **K**) and protein expression (**L**–**N**) level of Shh targets (**J**, **L**, and **M**) and RNF220 (**K**, **L**, and **N**) in the indicated tumor xenografts. IB immunoblot, WT wild-type, CA Smurf1 E3 ubiquitin ligase-defective mutant, CG Smurf2 E3 ubiquitin ligase-defective mutant, S1 Smurf1, S2 Smurf2, ns not significant. *p* > 0.05, *p < 0.05, and **p < 0.01.

Smurf1 and Smurf2 are HECT-type ubiquitin E3 ligases mediating protein ubiquitination. Therefore, we determined whether the growth-inhibiting activity of Smurf1 or Smurf2 is associated with their E3 ubiquitin ligase activity. Growth curves were generated for Daoy and UW228 cells expressing wild-type or ubiquitin E3 ligase-defective Smurf1 or Smurf2 and compared to controls in vitro. Growth curves generated over 7 days revealed that, relative to controls, expression of wild-type Smurf1 or Smurf2, but not their E3 ligase-defective mutant, significantly decreased cell proliferation of Daoy and UW228 cells (Fig. 6A and Supplementary Fig. 5A). Consistently, decreased EdU incorporation in wild-type, but not E3 ligase-defective mutant, expressing cells was observed (Fig. 6B, C and Supplementary Fig. 5B, C). We then examined the colony formation capacity of Daoy and UW228 cells stably transfected with wild-type or E3 ligase-defective mutants of Smuf1 or Smurf2. The results showed that expression of wild-type Smurf1 or Smurf2, but not their mutants, reduced colony formation capacity in the two cell lines (Fig. 6D–F and Supplementary Fig. 5D–F).

These data were further confirmed in xenograft models. During a 2-month period, Smurf1- and Smurf2-overexpressing cells grew significantly slower than the control cells, while the growth of cells expressing the E3 ligase-defective mutants was comparable to that of control cells (Fig. 6G). Consistently, the average weights and volumes of tumor xenografts from wild-type Smurf1- and Smurf2-expressing cells, but not the E3 ligase-defective mutants, were significantly reduced compared with those from the controls at Day 60 (Fig. 6H, I). Accordingly, the mRNA and protein expression levels of Shh targets, including Gli1, Ptch1 and Hhip1, were also reduced in xenografts from cells overexpressing wild-type Smurf1 and Smurf2 but not the E3 ligase-defective mutants (Fig. 6J, L, and N). Notably, only the protein level of RNF220 was downregulated in wild-type Smurf1- and Smurf2-expressing xenografts (Fig. 6K, L, and N). Collectively, these data suggest that Smurf1 and Smurf2 play an important role in driving Shh signaling-mediated tumor growth, possibly through the ubiquitin E3 ligase activity.

### Correlation of RNF220 and Smurf1 or Smurf2 protein expression in human clinical MB samples

**Fig. 7:**
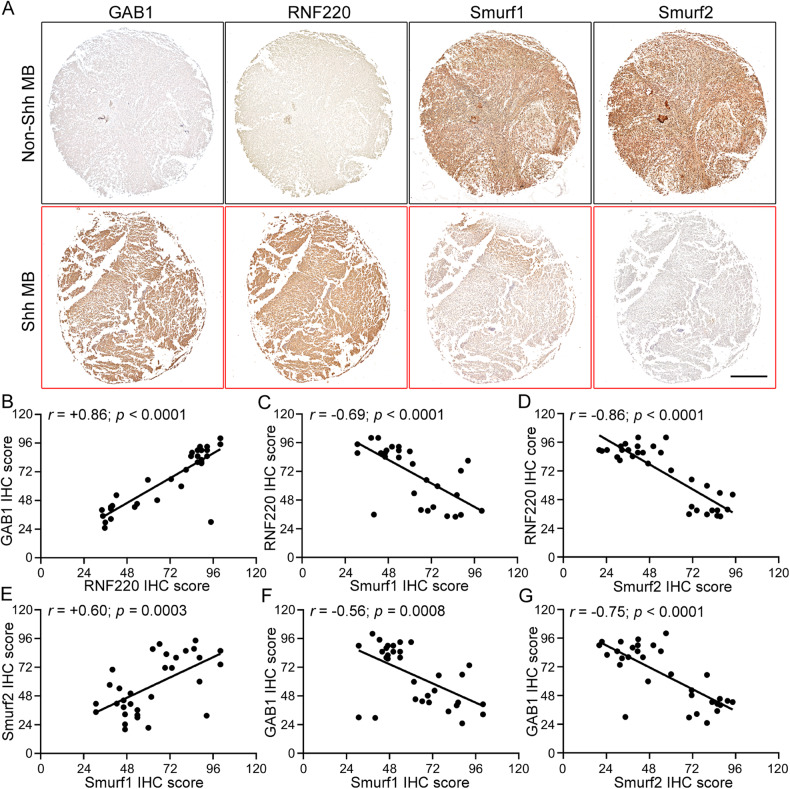
Protein expression correlation analyzes among GAB1, RNF220, Smurf1, and Smurf2 in human clinical medulloblastoma samples. **A** Representative immunohistochemical images of clinical Shh- and non-Shh medulloblastoma samples with the indicated antibodies. Scale bar, 5 mm. **B**–**G** Statistical analysis of the correlation among GAB1-RNF220 (**B**), Smurf1-RNF220 (**C**), Smurf2-RNF220 (**D**), Smurf1-Smurf2 (**E**), Smurf1-GAB1 (**F**), and Smurf2-GAB1 (**G**) based on the immunohistochemical scores, Pearson product-moment correlation coefficient analysis was used for the statistics. MB medulloblastoma, IHC immunohistochemical staining.

To test the clinical relevance of the Smurf1- or Smurf2-RNF220 axis in MB, we examined the expression levels of RNF220, Smurf1 and Smurf2 proteins in human clinical MB specimens using immunohistochemistry (Fig. 7). Shh-MB was identified by high GAB1 expression [35]. As previously reported, the expresssion of RNF220 correlated well with that of GAB1, confirming the function of RNF220 during Shh-MB progression (Fig. 7A, B) [12]. A strong negative correlation was observed between Smurf1 or Smurf2 and RNF220 in these MB samples (Fig. 7A, C, and D). Notably, negative correlations between GAB1 and Smurf1 or Smurf2 were observed (Fig. 7A, F, and G), confirming the downregulated expression of Smurf1 and Smurf2 in Shh-MB and supporting a tumor-suppressive role of Smurf1 and Smurf2 during Shh-MB progression. Notably, by analyzing the transcriptomic dataset (GSE85217) of 763 human MB samples using the R2 platform (Genomics Analysis and Visualization Platform, http://r2.amc.nl), neither positive nor negative correlation between RNF220 and Smurf1 or Smurf2 at their mRNA levels was found (Supplementary Fig. 6). Collectively, these data supports a regulation of RNF220 by Smurf1 and Smurf2 occurs at a posttranslational level through the degradation-mediated mechanism as described above in human Shh-MB.

## Discussion

In this study, we reported the involvement of Smurf1 and Smurf2, two HECT-type ubiquitin E3 ligases, in regulating the protein stability of RNF220 and, thus, Shh signaling during Shh-MB progression. Our work advances the understanding of the function of Smurf1 and Smurf2 in tumor biology and provides new potential diagnostic and drug targets for Shh-MB.

In addition to its canonical function in TGF-β/BMP signaling through targeting Smads [28, 29], Smurf1 and Smurf2 elicit diverse roles in the regulation of numerous signaling pathways through an array of different targets, including Wnt/PCP [36], hedgehog [15–18], hippo [37], and NF-κB signaling [38]. In the Shh signaling pathway, Smurf1 and Smurf2 have been shown to modulate Shh signaling by mediating ubiquitination and, thus, the trafficking or turnover of Ptch1 and Smo reciprocally [15–18]. Our data established a new role of Smurf1 and Smurf2 in Shh signaling through targeting RNF220, which was reported to positively regulate Shh signaling in our previous study [12]. Together with previous studies, we propose Smurf1 and Smurf2 as bivalent modulators of Shh signaling.

Smurf1 and Smurf2 are considered to act as both promoters and suppressors under different conditions through regulating certain targets involved in cancer progression [14]. Here, we reported a tumor-suppressive role for Smurf1 and Smurf2 during Shh-MB progression, which was proven by the following evidence. First, overexpression of either Smurf1 or Smurf2 inhibited the proliferation, colony formation and xenograft growth of Shh-MB cells (Fig. 6 and Supplementary Fig. 4). Second, consistently, knockdown of either Smurf1 or Smurf2 accelerated cell growth, and promoted the colony formation ability and xenograft growth of Shh-MB cells (Fig. 5 and Supplementary Fig. 3). Third, the protein expression of either Smurf1 or Smurf2 showed a negative correlation with that of GAB1, a marker for Shh-MB, in human clinical samples (Fig. 7) [35]. Previous analysis of gene expression using publicly available MB datasets also showed that expression of both Smurf1 and Smurf2 was downregulated in Shh-MB samples, compared to other subgroup MB samples [19, 20]. Consistently, we validated the reduced expression of Smurf1 and Smurf2 in the Ptch1^±^ mouse spontaneous orthotopic MB model (Fig. 4A–C). Previous reports have documented that Smurf2 heterozygotes are prone to spontaneous tumors in mice [39], and although Smurf2 knockout mice are relatively normal in their early life, with age, these mice develop various tumors in many tissues, including blood, lung, and liver [40]. Taken together, these findings highlight a tumor-suppressive role for Smurf1 and Smurf2 in Shh-MB.

Our previous study proposed that elevated RNF220 protein levels are achieved by a translational or posttranslational mechanism [12]. Here, we provide evidence that RNF220 protein is directly targeted and polyubiquitinated by Smurf1 and Smurf2, and the corresponding Smurf1 and Smurf2 downregulation is responsible for RNF220 upregulation in Shh-group MB. Whether the Smurf1- and Smurf2-RNF220 interaction and regulation are specific for Shh-MB needs further investigation. Our recent study also reported that ZC4H2 acts as a stabilizer for RNF220 protein by attenuating its polyubiquitination and that the Shh-RLIM-ZC4H2 axis is responsible for RNF220 stabilization in Shh-MB [22, 41]. Whether ZC4H2 affects Smurf1- and Smurf2-mediated polyubiquitination of RNF220 remains to be studied. ZC4H2 and Smurfs potentially compete to interact with the same RNF220 protein pool; alternatively, ZC4H2 interaction might block the sites for Smurf1- or Smurf2-mediated polyubiquitination. In addition, how Smurf1 and Smurf2 are downregulated in Shh-MB requires further analysis.

## Supplementary information

Supplementary Information

Reproducibility Checklist

## Data Availability

The materials used and/or analyzed during the current study are included in this published article and its Supplementary Information files. Additional data supporting the findings of this study are available from the corresponding authors upon reasonable request.
