# Supplementary material for: Smurf1 and Smurf2 mediated polyubiquitination and degradation of RNF220 suppresses Shh-group medulloblastoma
Source: Cell Death Dis. 2023 Aug 3;14(8):494. doi: 10.1038/s41419-023-06025-2 (PMC10400574; doi:10.1038/s41419-023-06025-2)
Supplement: Supplementary file 1 — Supplementary Information [file 41419_2023_6025_MOESM1_ESM.docx]

**Supplementary Information**

**Supplementary Figures list:**

Supplementary Figure 1, related to Figure 2.

Supplementary Figure 2, related to Figure 4.

Supplementary Figure 3, related to Figure 4.

Supplementary Figure 4, related to Figure 5.

Supplementary Figure 5, related to Figure 6.

Supplementary Figure 6, related to Figure 7.

**
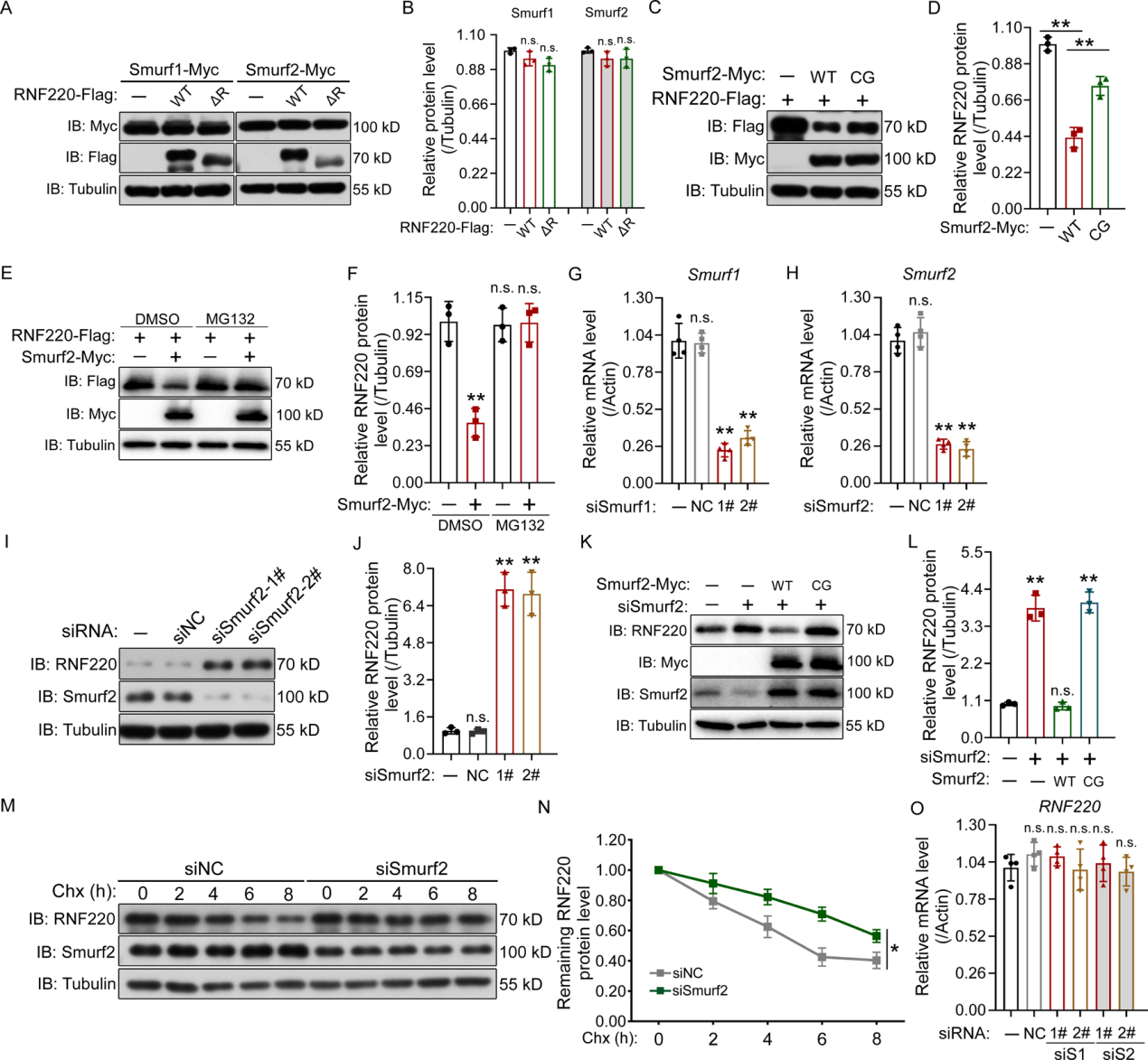
**

**Supplementary Figure 1, related to Figure 2. Smurf2 regulates the protein stability of RNF220.** (**A, B**) Western blot assays showing the protein level of Smurf1 or Smurf2 when co-expressed with wild-type or RING domain deleted RNF220 in HEK293 cells. HEK293 cells were transiently transfected with the indicated plasmids encoding Flag-tagged RNF220 or Myc-tagged Smurf1 or Smurf2, and 48 hours later, cells were harvested and analyzed by Western Blot. (B) Bar graphs, overlaid with the actual data points, show the relative protein levels (mean ± SEM) normalized against the level of corresponding α-Tubulin. The control was set to 1. (**C, D**) RNF220 protein was destabilized by overexpression of wild-type Smurf2 (C), but not its E3 ubiquitin ligase defective mutant. Flag-tagged RNF220, myc-tagged wild-type or E3 ubiquitin ligase defective Smurf2 plasmids were transfected into HEK293 cells as indicated. After 48 hours, cell lysates were analyzed by Western Blot. (D) Bar graphs, overlaid with the actual data points, show the relative expression (mean ± SEM) normalized against the corresponding α-Tubulin. The control was set to 1. (**E, F**) Western Blot analysis showing the protein level of RNF220 when co-expressed with Smurf2 in presence of MG132 or not. (F) Bar graphs, overlaid with the actual data points, show relative RNF220 protein expression (mean ± SEM) normalized against the corresponding α-Tubulin. The control was set to 1. (**G, H**) Real-time RT-PCR assays showing the mRNA level of Smurf1 (G) or Smurf2 (H) in HEK293 cells transfected with the indicated siRNAs against Smurf1 (G) or Smurf2 (H). (**I, J**) Western Blot results showing the effects of Smurf2 knockdown on RNF220 protein level in HEK293 cells. Cells were transfected with the indicated siRNAs, and 72 hours later, cells were harvested for Western Blot analysis. (J) Bar graphs, overlaid with the actual data points, show the relative expression (mean ± SEM) of RNF220, normalized against the corresponding α-Tubulin. The control was set to 1. (**K, L**) Western Blot analysis showing the protein level of endogenous RNF220 when wild-type or E3 ubiquitin ligase activity defective Smurf2 was co-expressed with siRNAs against Smurf2 in HEK293 cells. The statistics of the result was shown in (L) with α-Tubulin as a loading control, and the control was set to 1. (**M, N**) Effect of Smurf2 knockdown on the stability of endogenous RNF220 in HEK293 cells. Cells were transiently transfected with the indicated siRNAs. At 72 hours post-transfection, cycloheximide was added to all samples, and the cells were then harvested at the time points indicated. Protein level of RNF220 was determined by Western Blot with an anti-RNF220 antibody. The relative levels of RNF220 were quantified densitometrically and normalized against α-Tubulin. (N) The statistics showing the average of three independent experiments. (O) Real-time RT-PCR assays showing the mRNA level of RNF220 in HEK293 cells transfected with the indicated siRNAs against Smurf1 or Smurf2. IB, immunoblot; WT, wild-type; ΔR, RING domain deletion; CG, Smurf2 E3 ubiquitin ligase defective mutant; NC, negative control; Chx, cycloheximide; n.s., *p* > 0.05, not significant; *, *p* < 0.05; and **, *p* < 0.01.


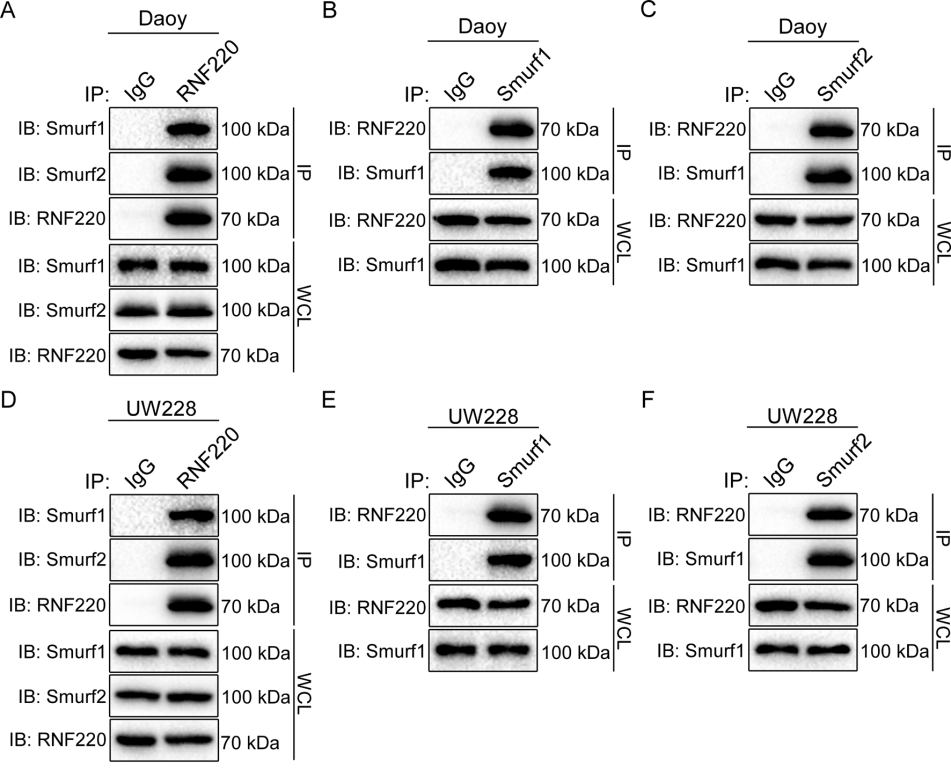


**Supplementary Figure 2, related to Figure 4. RNF220 interacts with Smurf1 and Smurf2 in Shh-MB cells.** *In vivo* co-IP analysis showing the interaction between RNF220 and Smurf1 or Smurf2 in Daoy (A-C) and UW228 cells (D-F)**.** IgG was used as a negative control for IPs. IB, immunoblot; IP, immunoprecipitation; WCL, whole cell lysate.


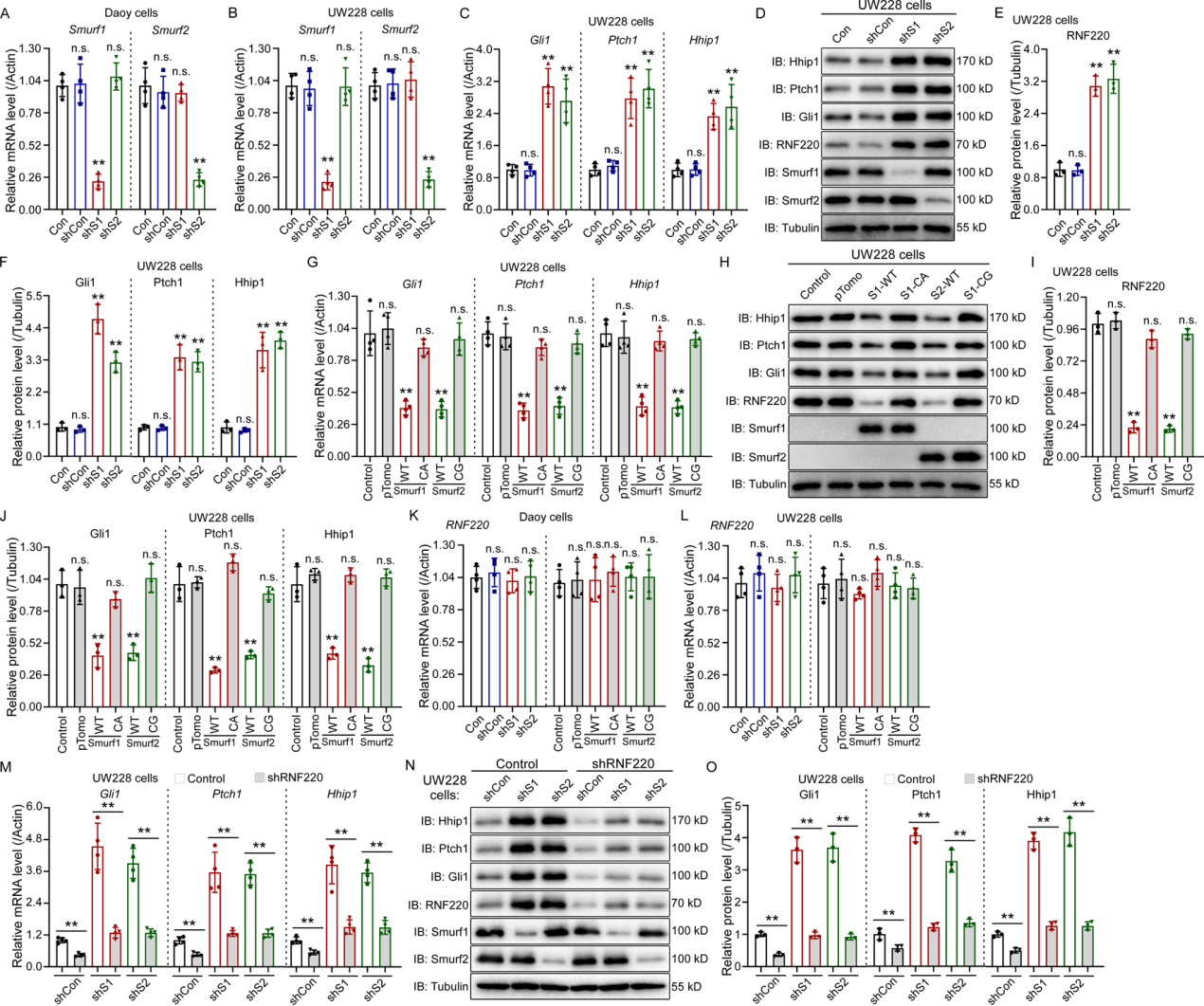


**Supplementary Figure 3, related to Figure 4.** **Smurf1 and Smurf2 regulate Shh signaling through RNF220 in UW228 cells.** (**A, B**) Real-time PCR assays showing the mRNA levels of Smurf1 and Smurf2 in Daoy (A) or UW228 (B) cells stably transfected with shRNAs against Smurf1 or Smurf2. (**C-F**) Real-time PCR (C) and Western Blot (D-F) assays showing the expression levels of RNF220 and Shh targets, including Gli1, Ptch1, and Hhip1, in UW228 cells stably transfected with the indicated shRNAs against Smurf1 or Smurf2. The statistics showing relative indicated protein levels normalized against the corresponding α-Tubulin level was shown in (E, F), and the respective controls were set to 1. (**G-J**) Real-time PCR (G) and Western Blot (H-J) assays showing the expression levels of RNF220 and Shh targets, including Gli1, Ptch1, and Hhip1, in UW228 cells overexpressed with wild-type or E3 ubiquitin ligase inactive Smurf1 or Smurf2. The statistics showing relative indicated protein levels normalized against the corresponding α-Tubulin level was shown in (I, J), and the respective controls were set to 1. (**K, L**) Real-time PCR assays showing the mRNA level of RNF220 in Daoy (K) or UW228 (L) cells transfected with the indicated shRNAs or overexpression plasmids. The respective controls were set to 1. (**M-O**) Real-time PCR (M) and Western Blot (N, O) assays showing the expression levels of Shh targets, including Gli1, Ptch1, and Hhip1, in UW228 cell stably transfected with the indicated shRNAs against Smurf1, Smurf2 or RNF220. The statistics showing relative indicated protein levels normalized against the corresponding α-Tubulin level was shown in (O), and the respective controls were set to 1. β-Actin was used as a loading control for realtime RT-PCR assays. IB, immunoblot; S1, Smurf1; S2, Smurf2; WT, wild-type; CA, Smurf1 E3 ubiquitin ligase defective mutant; CG, Smurf2 E3 ubiquitin ligase defective mutant; n.s., *p* > 0.05, not significant; and **, *p* < 0.01.


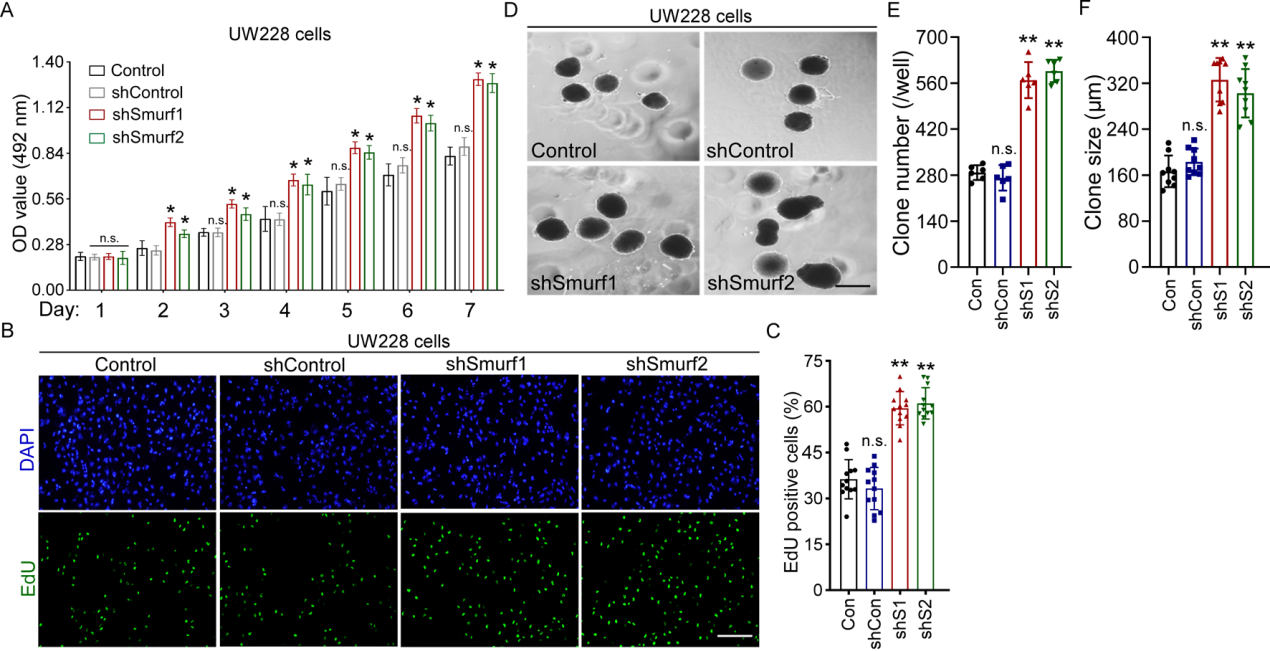


**Supplementary Figure 4, related to Figure 5. Smurf1 or Smurf2 knockdown accelerates cell proliferation in UW228 cells.** (**A**) Growth curve for control, Smurf1 or Smurf2 knockdown UW228 cell line, revealed by MTS assays. (**B, C**) EdU incorporation assay to evaluate DNA synthesis and proliferation rates of UW228 cells when Smurf1 or Smurf2 was knocked-down. Scale bar, 50 µm. Quantification of EdU assay results was shown in (C). (**D-F**) Soft agar colony formation assays for the indicated UW228 cell line. Scale bar, 120 µm. Quantification of colony number and size was shown in (E, F). S1, Smurf1; S2, Smurf2; IB, immunoblot; n.s., *p* > 0.05, not significant; and **, *p* < 0.01.


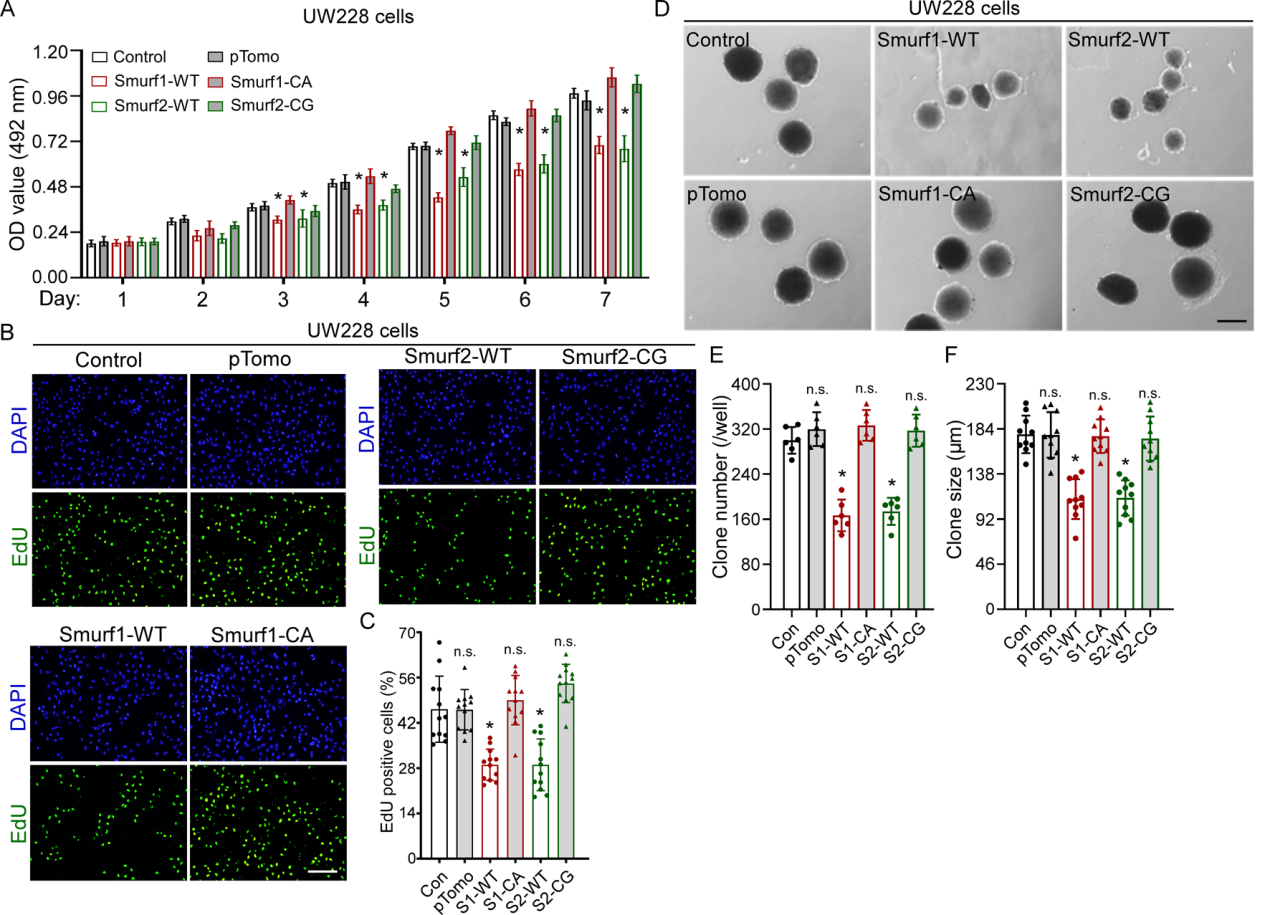


**Supplementary Figure 5, related to Figure 6. E3 ubiquitin ligase activity is required for cell proliferation inhibition by Smurf1 or Smurf2 overexpression in UW228 cells.** (**A**) Growth curve for control, wild-type or E3 ubiquitin ligase defective Smurf1 or Smurf2 overexpressed UW228 cell line, revealed by MTS assays. (**B, C**) EdU incorporation assay to evaluate DNA synthesis and proliferation rates of UW228 cells when wild-type or E3 ubiquitin ligase inactive Smurf1 or Smurf2 was overexpressed. Scale bar, 50 µm. Quantification of EdU assay results was showed in (C). (**D-F**) Soft agar colony formation assays for the indicated UW228 cell line. Scale bar, 120 µm. The quantification of colony number and size was showed in (E, F). WT, wild-type; CA, Smurf1 E3 ubiquitin ligase defective mutant; CG, Smurf2 E3 ubiquitin ligase defective mutant; S1, Smurf1; S2, Smurf2; n.s., *p* > 0.05, not significant; and *, *p* < 0.05.


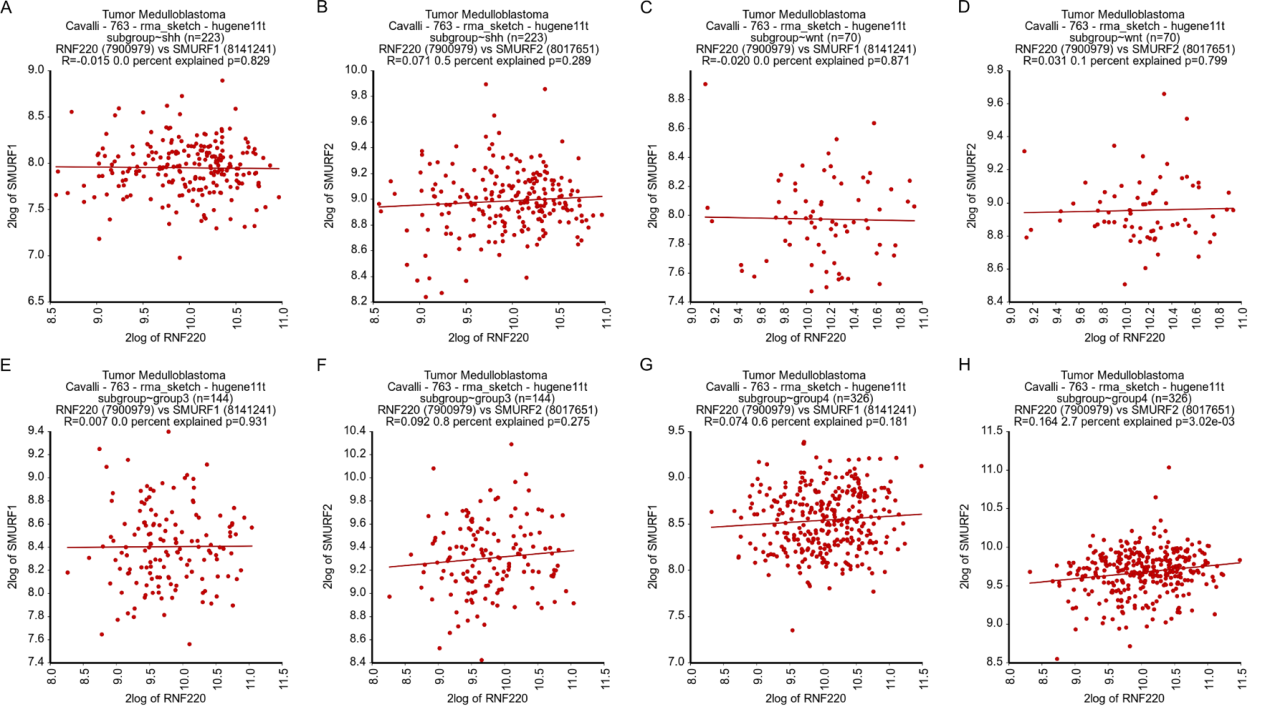


**Supplementary Figure 6, related to Figure 7. Correlations between RNF220 and Smurf1 or Smurf2 in the MB dataset (GSE85217) from R2: Genomics Analysis and Visualization Platform (http://r2.amc.nl).** The inverse correlation between RNF220 and Smurf1 or Smurf2 at mRNA level was not observed in any MB group. (A, B) Correlation between RNF220 and Smurf1 (A) or Smurf2 (B) in Shh-MB cohorts. (C, D) Correlation between RNF220 and Smurf1 (C) or Smurf2 (D) in Wnt-MB cohorts. (E, F) Correlation between RNF220 and Smurf1 (E) or Smurf2 (F) in Group3-MB cohorts. (G, H) Correlation between RNF220 and Smurf1 (G) or Smurf2 (H) in Group4-MB cohorts.
